# Orchestrating Teacher, Peer, and Self-Feedback to Enhance Learners’ Cognitive, Behavioral, and Emotional Engagement and Public Speaking Competence

**DOI:** 10.3390/bs14080725

**Published:** 2024-08-20

**Authors:** Tingting Liu, Vahid Aryadoust

**Affiliations:** 1 School of English Studies, Sichuan International Studies University, Chongqing 400031, China; tingting.liu@sisu.edu.cn; 2 National Institute of Education, Nanyang Technological University, Singapore 637616, Singapore

**Keywords:** engagement, oral presentation, peer feedback, public speaking competence, self-feedback, teacher feedback

## Abstract

Previous research on providing feedback on public speaking has investigated the effectiveness of feedback sources, namely teacher feedback, peer feedback, and self-feedback, in enhancing public speaking competence, predominantly individually. However, how these sources of feedback can be collectively harnessed to optimize learner engagement and public speaking performance still warrants further investigation. Adopting a pre- and post-test quasi-experimental design, this study randomly assigned four classes to four feedback conditions: Group 1 received teacher feedback, Group 2 self-feedback and teacher feedback, Group 3 peer and teacher feedback, and Group 4 feedback from all three sources. Both student engagement, measured using the Public Speaking Feedback Engagement Scale (PSFES), and their public speaking performance ratings, assessed using the Public Speaking Competency Instrument (PSCI), were validated using Rasch analysis. The inferential statistics revealed that Group 3 showed significant improvements across nearly all three dimensions of engagement, whereas Group 2 experienced significant declines in all dimensions of engagement except behavioral engagement. Group 3 demonstrated significantly greater engagement gain compared to Groups 2 and 4, indicating the synergistic effect of peer and teacher feedback in contrast to the limited impact of self-feedback. Additionally, all groups demonstrated significant improvements except for Group 2, which showed significantly lower improvement compared to Group 4. The following correlation analysis identified a significant correlation between the gain of students’ behavioral engagement and the gain of public speaking performance, whereas such association was absent between cognitive or emotional engagement and public speaking competence. This study suggests that peer feedback should be preceded by group discussion and supplemented with teacher feedback in classes for enhancing the teacher–student dialog, while self-feedback should be conducted after class to improve student engagement and public speaking performance.

## 1. Introduction

Public speaking is an act of communication to a group of people in a structured and informed manner in order to inform, influence, or entertain the audience [1]. In the classroom context, feedback from different sources (i.e., teacher feedback, peer feedback, and self-feedback) serves as a critical link in shaping the learning trajectory of public speaking skills. Feedback, as Hattie and Timperley [2] put it, is “the information provided by an agent (e.g., teacher, peer, book, parent, self, experience) regarding aspects of one’s performance or understanding”, which aims at reducing the gap between actual and the desired performance (p. 81). Previous research has investigated the effectiveness of feedback sources on public speaking competence, mostly individually [3,4], while these feedback sources are often integrated in authentic classrooms [5,6]. The orchestration of the sources in classrooms to optimize learners’ public speaking performance remains under-researched.

To enhance the actual uptake of feedback, student engagement with feedback—a process from collecting, digesting, and reflecting to acting on feedback, whether immediately or latently—should be supported [7]. Investigating how students engage with feedback from different sources can help tailor feedback instructions that are more likely to be utilized by students [7,8]. However, the existing literature on student engagement with feedback in public speaking, especially comparative studies, remains limited.

Therefore, the present study aims to investigate the impact of different sources of feedback on student engagement and public speaking performance in authentic classroom settings, as well as the association between student engagement and their public speaking performance.

### 1.1. Peer Feedback, Self-Feedback, and Teacher Feedback on Public Speaking

Peer feedback is a communication process through which learners engage in dialogic evaluation of a performance by their peers using defined criteria [9]. Students view peer feedback as interactive [10], encouraging [11], and easy to relate to [12].

While peer feedback can be motivating, it is often criticized for lacking substance. Simpson et al. [10] reported that an overwhelming majority (71.1%) of the students regarded assessor feedback as more credible than peer feedback. This lack of confidence in the utility of peer feedback could be due to students’ tendency to provide complementary rather than critical feedback [11]. Similarly, Day et al. [13] found that students’ presentation skills improved irrespective of the quantity of peer feedback received or provided, which they ascribed to the prevalence of evaluative comments in contrast to the lack of constructive suggestions. Another reason may lie in the written form of peer feedback on online platforms, wherein limited amount of information and a lack of direct communication can hinder the understanding and uptake of feedback [14].

Oral peer feedback, due to its interactive nature, can elicit more information and allow direct and efficient understanding. Hung [15] addressed the problem of students’ perceived insufficiency of peer feedback by asking students to upload video-recorded oral feedback to their peers. Results from surveys and interviews indicated that students allocated more attention to the content when it was oral rather than textual. However, some students also reported that they struggled to include specific points in their feedback. This draws attention to the importance of brainstorming with peers prior to offering peer feedback.

Group discussion following students’ speaking practice is an important prelude before providing peer feedback for speakers to have authentic speaking experience and for the plenary class to associate the performance with the criteria. Students commented that presenting in class and discussing the performance with peers allowed for more authentic speaking practice [4] and they found it interesting [6]. Additionally, peer feedback as a group adds to pooled credibility and reduces concerns of offending peers during one-on-one peer feedback. Banister [5] observed a lively group discussion after student presentations and observed students’ enthusiasm in providing peer feedback in a face-to-face group peer-feedback classroom activity.

In addition to the external source of feedback from peers, self-feedback, as an internal feedback source, typically involves activities such as watching one’s own presentation videos, evaluating one’s performance using provided rubrics, and writing reflective pieces [16,17]. One recurring benefit of self-feedback lies in its usefulness in raising students’ awareness of the discrepancies between their current and desired performance [16,17,18]. However, students commented that they struggled to maintain objectivity about their own speaking performance and often exhibit excessive self-criticism [17].

To address the subjectivity challenge of using students’ self-feedback alone, external feedback by teacher or peers can serve as an anchor for their internal feedback. A case study by Carless [6] of an award-winning teacher’s pedagogy reported that the teacher would video-record students’ oral presentation, show extracts to the class, and then invite students to self-evaluate their performance, followed by peer feedback interspersed with comments and questions from the teacher to provoke deep thinking.

In addition to peer feedback as an external source to self-feedback, in studies in authentic classroom contexts, teacher feedback is typically provided as a summary of the feedback loop [4,18]. Students expressed their expectation that teacher feedback be included in class as a more authoritative source [4,5]. This could be due to the modeling and facilitating role of the teacher as a professional source of feedback and the vicarious learning activated by the teacher’s feedback on a few speaking examples to the plenary class [19].

### 1.2. Comparative Studies of Peer, Self-, and Teacher Feedback on Public Speaking

A literature review showed that teacher feedback is typically provided as a summary of peer feedback [4,5] or interspersed with peer and self-feedback in authentic classroom settings [9], and each of the three sources has its advantages and drawbacks. An ensuing question is how effective these feedback sources relatively are in fostering desired public speaking performance.

In a (quasi-)experimental study, Murillo-Zamorano and Montaner [12] found that peer feedback resulted in better public speaking skills compared to teacher feedback. They observed heightened discussion and self-reflection triggered among the feedback group, attributing its relative effectiveness to students’ ability to empathize with their peers’ challenges and the clarity of the feedback message. However, only one rater was engaged in rating, which was not validated.

In contrast, other comparative studies have found teacher feedback to be more effective than peer or self-feedback in improving public speaking skills. De Grez et al. [20] found that among the three sources of feedback provided in written form on an online platform, the group receiving teacher feedback showed the greatest improvement (13%) compared to those receiving peer feedback (7.5%) and self-feedback (0.2%).

In another comparative study, van Ginkel [3] reported that the teacher feedback group achieved significantly higher oral presentation scores compared to the self-feedback group, peer feedback group, and the peer feedback group guided by a tutor. van Ginkel et al. [21] attributed the facilitative role of the teacher feedback to its high quality, as analyzed against the seven principles of effective feedback identified in a previous systematic review [22]. Contrastingly, no significant differences were observed among the four groups regarding their cognition of and attitude toward oral presentations. This suggests that students’ cognitive and emotional engagement with the different sources of feedback may not align with their behavioral engagement. However, little or no classroom-based research has compared the impact of different combinations of feedback sources on students’ public speaking performances and engagement.

### 1.3. Engagement with Feedback on Public Speaking

A pivotal factor contributing to the effectiveness of feedback is the active engagement of students in order to utilize it to its fullest potential [23]. Student engagement is a multi-dimensional construct comprising (1) behavioral, (2) cognitive, and (3) emotional engagement, and should be further defined and measured in specific contexts [24,25]. In the context of engagement with feedback in writing and public speaking, cognitive engagement with feedback refers to students’ investment in cognitive efforts, such as noticing and understanding feedback [26], strategizing to revise their texts [27], and employing metacognitive strategies to monitor and regulate their processing of feedback and subsequent revisions [28]. Students’ behavioral engagement with feedback can be observed through their revisions based on the feedback [24,28], reference to relevant materials [26], and practice of speech delivery [29]. Emotional engagement is reflected in students’ affective responses, including surprise, happiness, frustration, and more [30], such as valuing the worth of the feedback [31] and their appreciation of the use of feedback [28,29].

Laranjeira and Teixeira [32], using measurement scales, identified a correlation between student engagement and their academic performance in higher learning in general. However, some case studies on engagement with feedback in writing suggest that students’ cognitive, behavioral, and emotional engagement may not demonstrate synchronized growth or lead to desired performance. For example, Zheng and Yu [31] found that even though students reported positive emotional engagement, their cognitive and behavioral engagement was not as extensive and their behavioral engagement did not translate to enhanced language accuracy based on the evaluation of the students’ texts.

While several empirical studies have explored student engagement with teacher feedback in writing [26,31], there is a noticeable gap in the literature regarding student engagement with feedback in public speaking or oral presentations (e.g., [29]), particularly in comparison with peer and self-feedback. Additionally, the existing studies of engagement with feedback in writing and speaking have primarily relied on case studies, and further research is warranted to examine the generalizability of these results.

### 1.4. The Present Study

The present study aims to address research gaps for feedback in public speaking by contributing to this stream of research in the following ways. First, previous studies have primarily focused on written peer and self-feedback provided to students’ speech or speech videos through online platforms [4,11]. However, research suggests that feedback offered through interactive face-to-face dialog leads to greater knowledge acquisition and elaboration compared to written feedback without dialog [14,23]. This classroom-based study seeks to afford students with authentic public speaking experiences and opportunities for timely and direct dialog with their teachers and peers.

Second, the existing studies have compared teacher feedback with other feedback sources individually. Ajjawi et al. [14], in a systematic review, highlight the importance of relatedness to the teacher in the feedback for desired learning outcomes, and teacher feedback is typically incorporated with peer or self-feedback in authentic classroom instructions (e.g., [5,6]). Therefore, this study aims to compare the effect of teacher feedback combined with other feedback sources on student engagement and public speaking performance.

Finally, while case studies have explored engagement with feedback in writing skills (e.g., [26,31]) and oral presentations (e.g., [29]), the literature on engagement with feedback in public speaking and its impact on performance lacks comparative analyses. This study endeavors to validate a measurement scale for engagement and the ratings of students’ public speaking performance to support educators in leveraging the benefits of diverse feedback sources in their teaching practice. The specific research questions (RQs) are listed as follows:

RQ1: How do students engage with teacher feedback, peer feedback, and self-feedback cognitively, behaviorally, and emotionally?

RQ2: How do the three feedback sources impact students’ public speaking performance?

RQ3: How does students’ cognitive, behavioral, and emotional engagement with feedback correlate with their public speaking performance?

## 2. Methodology

### 2.1. Participants and Context

This study was conducted in an English public speaking course offered to second-year English majors at a university in China. A total of 98 students from four intact classes (21 males and 71 females, age 19–20) voluntarily participated in this study. The participants scored above 110 out of 150 points in English on the College Entrance Examination in China, which corresponds to the B1 to C1 band in the Common European Framework of Reference. The four classes had similar gender ratios, with male participants comprising 20.8% in Group 1, 20.0% in Group 2, and 20.8% in Group 3 and Group 4. All groups share the same age range of 19–20. All students were enrolled in the same compulsory major courses and had the same instructor for English public speaking course, ensuring minimal discrepancy in their English exposure within the classroom setting.

This course spans an 18-week semester, with two 45 min sessions separated by a 10-min break each week. Throughout the semester, each student prepared eight 3-min speeches before class and delivered three speeches in class, with different speakers each week due to the class’s time constraints. This course encompassed three progressive stages: (1) instruction on public speaking skills accompanied by student practice and teacher feedback, (2) training students to provide feedback, and (3) student practice with providing and receiving feedback. The intervention was implemented during the third phase to ensure that students had adequate public speaking and feedback skills.

### 2.2. Instruments

*Public Speaking Feedback Engagement Scale (PSFES)*. As students’ self-report is listed as one of the five major methods for measuring engagement [33], we developed the Public Speaking Feedback Engagement Scale (PSFES) to measure student engagement with feedback (see Appendix A.1). For the scale’s development, the authors firstly listed a pool of 16 statements based on the conceptualization of engagement (e.g., [24,25]), followed by consultation with four students and three public speaking instructors who have 10–15 years of teaching experience in public speaking for refinement. The finalized 15-item 5-point Likert scale operationalizes engagement with feedback on public speaking as follows:

- Cognitive engagement (items 1–6) is indicated by students’ thinking processes: understanding and reflecting on the feedback, recognizing their strengths and weaknesses, strategizing revisions, and monitoring their progress against the feedback when preparing subsequent speeches.
- Behavioral engagement (items 7–12) is manifested through actions taken by students in response to the feedback: taking notes on the feedback, discussing feedback with peers or teachers, revising speeches, organizing speech structure, searching for supporting evidence, and practicing speech delivery.
- Emotional engagement (items 13–15) is reflected by students’ subjective feelings: the extent to which they enjoy receiving or providing feedback, find it useful, and look forward to engaging in the feedback process. The instrument was validated using Rasch measurement (see Section 2.5. Data Analysis for the validation of the PSFES).

*Public Speaking Competency Instrument (PSCI)*. Student’s public speaking performance was rated by four raters using the PSCI, a 5-point Likert scale with 20 items categorized into five subscales: introduction, body, conclusion, delivery, and global competence [see validation evidence of the rubrics in 34]. The introduction items (1–3) evaluate the attention-getter, thesis statement, and transition from the introduction to the body. The body items (4–8) assess the main points, pattern of organization, adequacy, attractiveness, and appropriacy of the supporting materials. The conclusion items (9–12) examine the conclusion discourse marker, review of main points, and memorability of the closing remark. The delivery items (13–19) examine the speaker’s rate of speaking, volume, gestures, eye contact, confidence, and expressiveness of voice and body language. The global item (20) appraises the speaker’s overall competence [34]. The ratings based on the rating scale were validated using Many-facet Rasch measurement (MFRM) (see Section 2.5. Data Analysis for validation of the ratings).

*Feedback sheet*. We designed an 18-question feedback sheet based on the PSCI to help students organize their feedback and relate performances to specific criteria. Questions 1–3 (introduction) focus on the speaker’s use of an attention-getter, articulation of their thesis statement, and the transition to body. Questions 4–7 (body) evaluate the clarity of the main points, the logical organization of ideas, the adequacy of supporting evidence, and the coherence between points. Questions 8–10 (conclusion) examine whether the speaker reviewed the thesis or the main points, crafted a memorable ending, and included a compelling call to action. Questions 11–17 (delivery) assess the speaker’s use of voice and body language to engage the audience. Lastly, question 18 considers the audience’s overall impression of the speech (see Appendix A.2).

### 2.3. Intervention

**Figure 1:**
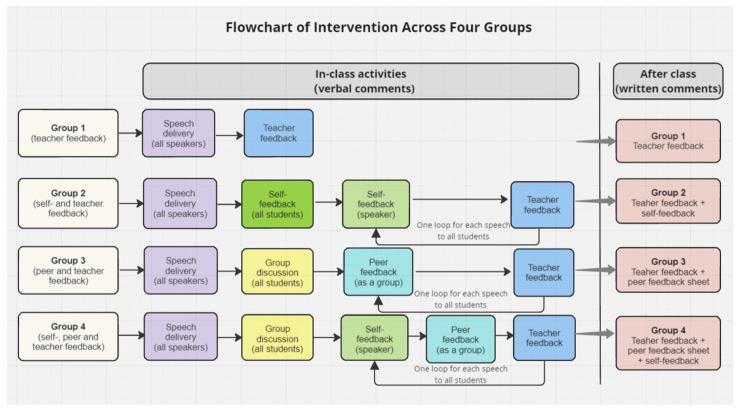
A flowchart of the intervention across four groups.

The intervention spanned four weeks, during which all students were required to prepare four 3-min speeches on four distinct topics (one for each week) and deliver one speech in class, with six students delivering their speeches in class each week due to the class’s time constraints. While each student was individually critiqued on their classroom presentation for once, the feedback was given to the entire class during the plenary session, focusing on four distinct topics to foster vicarious learning [19,35]. After students delivered their speeches, which were video-recorded using a Logitech C930e business webcam connected to a laptop to replay during feedback [19], the four intact classes were randomly assigned to the four feedback conditions: (1) teacher feedback, (2) self- and teacher feedback, (3) peer and teacher feedback, and (4) self-, peer, and teacher feedback (see Figure 1 for a flowchart of the intervention).

*Group 1 (teacher feedback)*. For each speech, the teacher provided a comprehensive 10 min of feedback to the plenary class on the structure, content cohesion, incorporation of evidence, effective linking of ideas with evidence, and speech delivery based on the 18-question feedback sheet. During feedback, the teacher replayed video extracts of students’ speeches to highlight certain behaviors.

*Group 2 (self- and teacher feedback)*. After being inspired by their peers’ speeches, all students engaged in a 15-min self-evaluation based on the feedback sheet to allow time for adequate self-critique [17], and the speakers’ videos were sent to them individually via QQ for reference. Next, each speaker gave a 3-min verbal self-critique to the class, followed by a 5-min teacher feedback while replaying the video extracts of the speeches. The teacher acknowledged and briefly discussed the points highlighted in students’ self-critiques, while inviting all students to revisit points that were not thoroughly addressed. After class, the speakers were required to watch their own videos and write a 200-word self-critique to further solidify their learning.

*Group 3 (peer and teacher feedback)*. Following students’ speech delivery, the classes were divided into six smaller groups to engage in a 20-min group discussion on all speakers’ performances and each speaker was invited to join one group. Video-recordings of all speeches were posted on the class QQ group chat for reference during group discussions. Each group took notes on the feedback sheet in preparation for providing constructive peer feedback. Next, for each speech, one group provided 3 min of verbal feedback to the entire class, while additional input from other groups was encouraged. Following peer feedback, the teacher provided 5 min of feedback on each speech, during which they provided clarification, emphasized agreement with students’ peer feedback, and elaborated on any missed points while replaying segments of the speech videos as needed. After class, the students’ feedback sheets were given to the speakers for reference.

*Group 4 (self-, peer, and teacher feedback)*. Similar to Group 3, the class first initiated a 20-min collaborative group discussion. Next, for each speech, the speaker was allotted a 2-min window to self-assess their performance, followed by 3 min of peer feedback on the speech given. This involved constructive dialogue between the speaker and feedback provider to prompt proper communication. Subsequently, the teacher provided 3 min of feedback on the speech, offering additional insights to complement students’ comments and addressing contentious issues if those arose during students’ discussion and feedback sessions, with video references when necessary. The speakers were provided the feedback sheets from their peers and were asked to watch their own speech videos and submit a 200-word written self-evaluation after class.

After class, the teacher distributed their written comments on the presenters’ speeches to all students for reference.

### 2.4. Procedures

This study was approved by the Institutional Review Board (IRB) of Nanyang Technological University. Ethical considerations were made by providing students with information regarding their right to anonymity and withdrawal at any time, and their consent for voluntary participation was sought. Before intervention, all students delivered a 3-min prepared persuasive speech on a given topic and completed the PSFES as pre-tests. After four weeks of intervention, students then completed both post-tests. Students delivered both of their pre-test and post-test speeches to the entire class and the instructor in the classroom, which were video-recorded for later ratings. The topics for the two speeches were carefully selected by the instructor and raters from a collection of topics used in a prestigious Chinese national public speaking competition to maintain a consistent level of difficulty. We did not use the same topic for the pre- and post-test to ensure the evaluation of the students’ ability to transfer their public speaking skills across topics.

After the pre- and post-tests, four raters with 10–15 years of public speaking teaching and rating experience served as raters to assess the participants’ speeches. Before rating, the raters underwent training on the rubrics of the PSCI and rated 15 speeches for the pre-test and post-test, respectively. Many-facet Rasch measurement (MFRM) analysis was conducted to evaluate the quality of the sample ratings, and any discrepancies were resolved through discussion and re-rating. Subsequently, the raters rated all pre-test and post-test speeches, which added up to a total of 15,680 ratings (98 participants × 20 items × 2 tests × 4 raters).

### 2.5. Data Analysis

*Validating the PSFES*. To validate the PSFES, we conducted Rasch–Andrich Rating Scale Model (RSM) analyses for each of the three subconstructs for both the pre- and post-test, using the *Winsteps* software package, Version 5.1.0 [36]. Rasch analysis is robust for questionnaire validation with smaller sample sizes, with a minimum requirement of 30 respondents [37]. For quality control, we examined (1) the unidimensionality of the subscale, (2) the item’s and people’s reliability and separation, (3) the item fit statistics, and (4) the scoring categories and thresholds [38].

First, unidimensionality is a precondition for Rasch measurement in that a scale should measure a single construct it intends to measure, which is indicated by the largest factor of the residuals, with the 1st contrast value lower than 2.0 indicating unidimensionality [39,40]. Second, high item reliability (>0.8) and separation (>2.0) demonstrate the items’ capability to differentiate low and high responses to items, and high person reliability indicates a wide range of responses among participants [39,40]. Third, item infit and outfit mean square (MnSq) are two statistics to detect erratic patterns in the item responses. Infit MnSq is sensitive to abnormal responses near a person’s ability, while outfit MnSq detects erratic patterns in the item responses distant from the respondents’ ability, with MnSq values between 0.5 and 1.5 suggesting good item fit [39]. Lastly, a category threshold is the point on the latent variable where someone has an equal probability of choosing one particular category or the one below it [39]. The threshold difficulty measures should increase monotonically by at least 1.1 logits (log-odd units), and the MnSq values should fall between 0.5 and 1.5 [39,40].

*Validating ratings of speeches*. We subjected the ratings of the pre- and post-test to MFRM, using the *Facets* computer program, Version 3.83.2 [41]. The MFRM adjusts the ratings for the effects of each of students’ speaking ability, rater severity, and item difficulty, and generates “fair averages”, which partial out the effect of these facets on the scores, thus eliminating the “bias” effect on the test scores [39,42]. The mathematical representation of this three-facet MFRM applied is given as follows:

(1) $$\log\frac{Pnijk}{Pnijk-1}=B_{n}-D_{i}-C_{j}-F_{k}$$

where *P_nijk_*_,_ is the probability that student speaker *n* with ability *B_n_* is rated by rater *j* of severity *C_j_* in category *k* of item *i* of difficulty *D_i_* as opposed to the probability *P_nijk_*_− 1_ of being rated in *k* − 1 category [43]. Thus, the probability of a student speaker receiving a score from a rater for each item depends on the student’s ability, item difficulty, scoring rubrics, and the rater’s severity/lenience [43].

*Inferential statistics.* For the inferential statistics for RQ1 and RQ2, we first attempted a mixed ANOVA. However, significant differences were found among the four groups in the pre-test, which rendered mixed ANOVA inappropriate in our case. We then conducted one-way ANOVA using the gain (post-test minus pre-test) of their engagement with feedback and public speaking performance. It should be noted that gain score analysis can effectively address the pre-existing differences in pre-test and post-test design [19,44,45,46]. The normality of residuals was examined and met. Post-hoc analysis was performed using Bonferroni correction when the homogeneity of variance was met, while Welch’s test and Games-Howell post-hoc test were used in cases where equal variance was violated. To determine if there were significant changes over time, we conducted paired samples *t*-test for both engagement and performance. Regarding RQ3, Pearson correlation was employed to examine the potential association between the gain of student engagement and the gain of their public speaking competence. All the inferential statistics were conducted using IBM SPSS Statistics, Version 26 [47].

## 3. Results

### 3.1. Rasch–Andrich Rating Scale Model Validation of the Public Speaking Feedback Engagement Scale (PSFES)

Table 1 presents the statistics for quality control for the PSFES. First, the analysis confirmed the unidimensionality of all three subscales in both the pre- and post-tests (first contrast < 2.0). Second, the results revealed high item reliability (0.86–0.97) and item separation (2.51–5.79) for all analyses, with the exception of the medium item reliability (0.7) and item separation (1.53) for cognitive engagement in the pre-test. Additionally, the findings showed medium person reliability for the pre-test (0.62–0.71) and post-test (0.57–0.74) with separation coefficients ranging from 1.15 to 1.67 for both tests, which does not indicate that the scale is flawed but that the participants were homogenous in their responses [39]. Third, all items showed good infit (0.70–1.48) and outfit range (0.67–1.56), with outfit for pre-test emotional engagement slightly above 1.5 (1.56), which does not degrade the measurement and was therefore kept in the analysis [39]. Lastly, all subscales showed monotonic increases between category measures, and the MnSq values ranged between 0.5 and 1.5, showing good fit with the RSM (see Appendix A.3 for item fit statistics and Appendix A.4 for category structure).

### 3.2. MFRM Validation of the Ratings Assigned to Students’ Public Speaking Performance

As shown in Table 2, there was no significant difference between the observed average (the average ratings) and the fair average (average ratings adjusted by the Rasch model), with low measurement error (0.18–0.22). Overall, no misfitting pattern emerged, as indicated by the infit range (0.54–1.47) and outfit range (0.54–1.45). The high separation for the pre-test (4.58) and post-test (5.71) indicated that the items effectively differentiated the students into approximately five or six levels. Additionally, the high person reliability for the pre-test (0.95) and post-test (0.97) suggests that the ratings are consistently reproducible (see Appendix A.5 for the fit statistics of the ratings).

### 3.3. The Impact of Feedback Sources on Student Engagement

Significant differences were identified among the four groups in the pre-test: cognitive engagement (*F*(3, 94) = 2.77, *p* = 0.046, partial η^2^ = 0.081), behavioral engagement (*F*(3, 94) = 3.45, *p* = 0.02, partial η^2^ = 0.099), emotional engagement (*F*(3, 94) = 3.24, *p* = 0.007, partial η^2^ = 0.119), and total engagement (*F*(3, 94) = 3.79, *p* = 0.004, partial η^2^ = 0.133). Therefore, gain score ANOVA was conducted for identifying differences among the four groups in the changes from pre-test to post-test (see Appendix A.6 for descriptive statistics for the pre-test and post-test engagement across the four groups).

**Figure 2:**
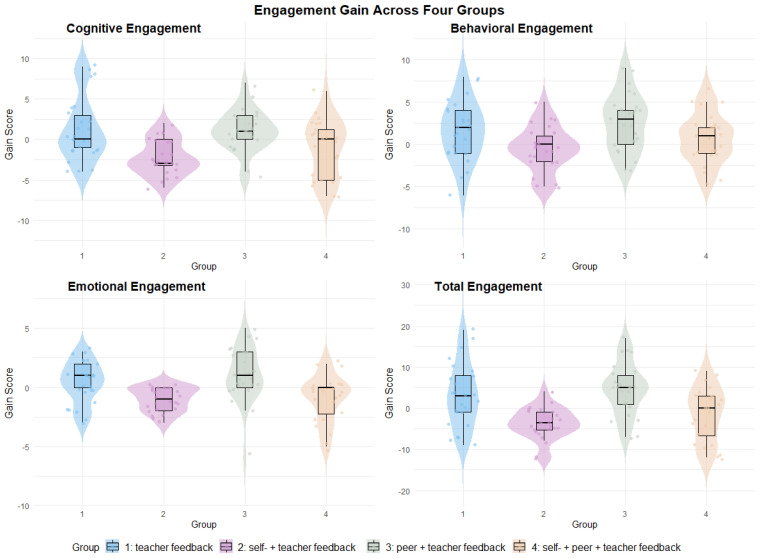
Engagement gain across four groups.

Table 3 presents the descriptive statistics for the gain in students’ cognitive, behavioral, emotional, and total engagement. Overall, Group 1 and Group 3 showed improvements in all types of engagement, whereas Group 2 and Group 4 experienced decreases in all four measures of engagement, except for behavioral engagement for Group 4 (see Figure 2).

**Figure 3:**
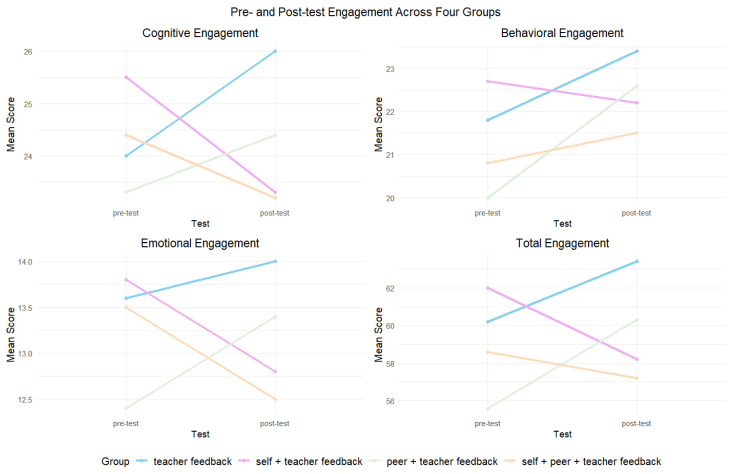
Pre- and post-test engagement across four groups.

Table 4 presents the results of the paired sample *t*-test for engagement across four groups. Regarding cognitive engagement, a significant decline was observed in Group 2 (*t*(23) = −5.206, *p* < 0.001, Cohen’s *d* = −1.063). For behavioral engagement, a significant improvement was noted in Group 1 (*t*(24) = 2.22, *p* = 0.036, Cohen’s *d* = 0.444) and Group 3 (*t*(24) = 3.82, *p* = 0.001, Cohen’s *d* = 0.764). Emotional engagement showed a significant decline in Group 2 (*t*(23) = −4.796, *p* < 0.001, Cohen’s *d* = −0.979) and Group 4 (*t*(23) = −2.361, *p* = 0.027, Cohen’s *d* = −0.482), whereas a significant increase was revealed in Group 3 (*t*(24) = 2.279, *p* = 0.032, Cohen’s *d* = 0.456). Finally, total engagement significantly decreased in Group 2 (*t*(23) = −4.867, *p* < 0.001, Cohen’s *d* = −0.995) but showed a significant increase in Group 3 (*t*(24) = 3.697, *p* = 0.001, Cohen’s *d* = 0.739) (see Figure 3).

**Figure 4:**
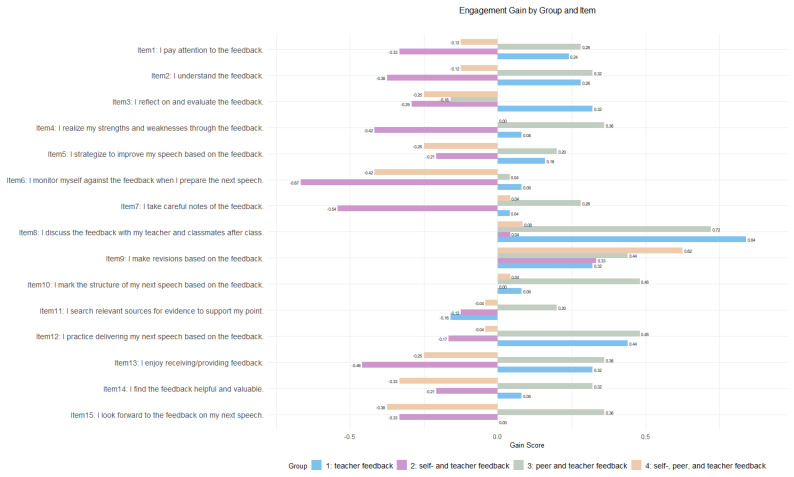
Engagement gain by group and item.

Specifically, regarding cognitive engagement, Group 1 and Group 3 made notable improvements in areas such as attention, comprehension, and reflection on feedback. They also reported better awareness of their strengths and weaknesses, as well as being more proactive in planning and monitoring their upcoming speeches. Similarly, Group 1 and Group 3 perceived an enhancement in emotional engagement, enjoying the feedback process, finding it useful, and anticipating it as opposed to the decrease in these aspects observed in Group 2 and Group 4. Behaviorally, all groups except Group 2 showed improvement by taking notes on the feedback, engaging in discussions out of class, revising their work, organizing the structure, and practicing speech delivery with Group 4 making the most revisions. However, except for Group 3, all other groups exhibited little improvement or even decreases in note-taking and searching for supporting evidence (see Figure 4 for responses to each item across groups).

The normality assumption was met for cognitive engagement (skewness = 0.349, kurtosis = 0.080), behavioral engagement (skewness = 0.034, kurtosis = −0.361), emotional engagement (skewness = −0.729, kurtosis = 1.595), and total engagement (skewness = 0.068, kurtosis = 0.080). Levene’s test of equality was violated for cognitive engagement (*p* = 0.011) and total engagement (*p* = 0.016), and therefore the Welch’s test and Games-Howell post hoc test were used for these two.

**Figure 5:**
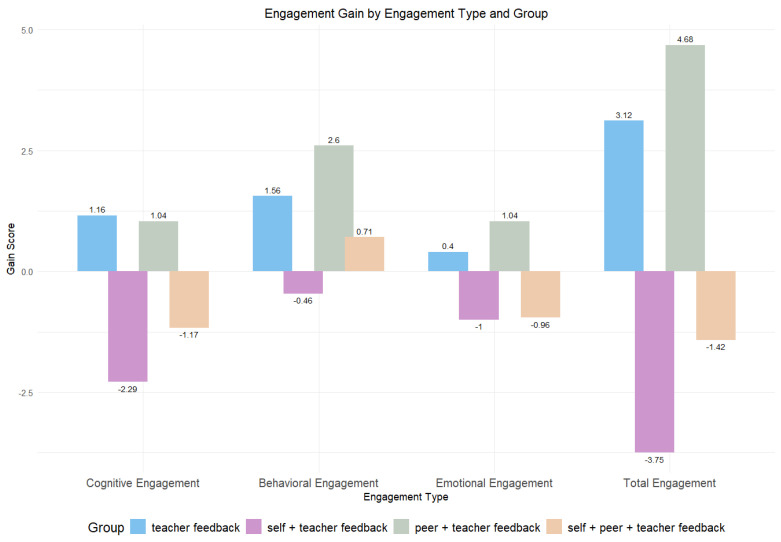
Engagement gain by engagement type and group.

A significant difference among groups was detected for all dimensions of engagement with medium-to-large effect sizes [44]. For cognitive engagement, the test revealed a significant difference [*Welch*’s *F*(3, 51.041) = 9.892, *p* < 0.001, partial η^2^ = 0.184], and the post hoc showed Group 2 was significantly lower than Group 1 (*p* = 0.003) and Group 3 (*p* < 0.001). Regarding behavioral engagement, a significant difference was observed [*F*(3, 94) = 4.102, *p* = 0.009, partial η^2^ = 0.116], and Group 2 was found to be significantly lower than Group 3 (*p* = 0.006). For emotional engagement, the findings revealed a significant difference (*F*(3, 94) = 7.523, *p* < 0.001, partial η^2^ = 0.194), and Group 3 was found to be significantly higher than Group 2 (*p* = 0.001) and Group 4 (*p* = 0.001). Finally, there was a significant difference in the total engagement (*Welch*’s *F*(3, 50.335) = 13.007, *p* < 0.001, partial η^2^ = 0.233). The post hoc test revealed that Group 3 had significantly higher engagement than Group 2 (*p* < 0.001) and Group 4 (*p* = 0.009), whereas Group 2 showed significantly lower engagement than Group 1 (*p* = 0.002) (see Figure 5).

### 3.4. Impact of Feedback Source on Students’ Public Speaking Performance

**Figure 6:**
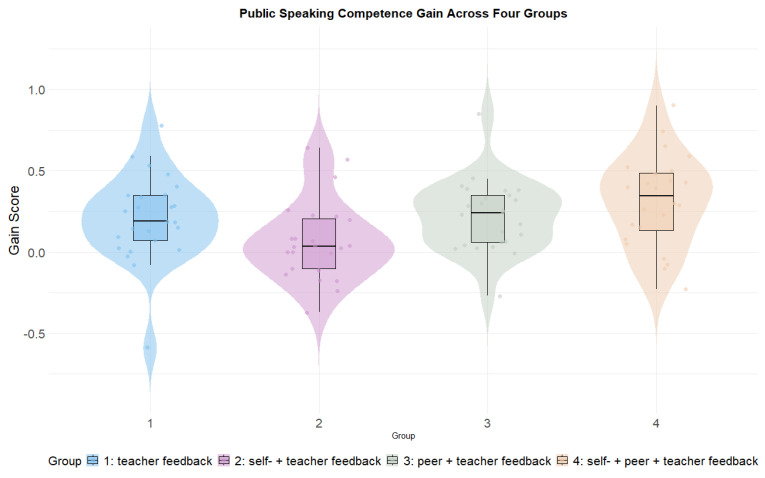
Public speaking competence gain across four groups.

A significant difference was identified in the pre-test public speaking performance (*F*(3, 94) = 4.81, *p* = 0.004, partial η^2^ = 0.133), and therefore the gain score ANOVA was examined. The residuals of gain of students’ fair measures were normally distributed (skewness = 0.050, kurtosis = 0.888) and the variance was equal across groups (*p* = 0.621). (see Appendix A.6 for descriptive statistics for the pre-test and post-test public speaking performance). All four groups made improvements, with Group 4 (*M* = 0.31, *SD* = 0.28) being the best-performing group, followed by Group 3 (*M* = 0.22, *SD* = 0.22), Group 1 (*M* = 0.21, *SD* = 0.27), and Group 2 (*M* = 0.07, *SD* = 0.24). (see Figure 6).

**Figure 7:**
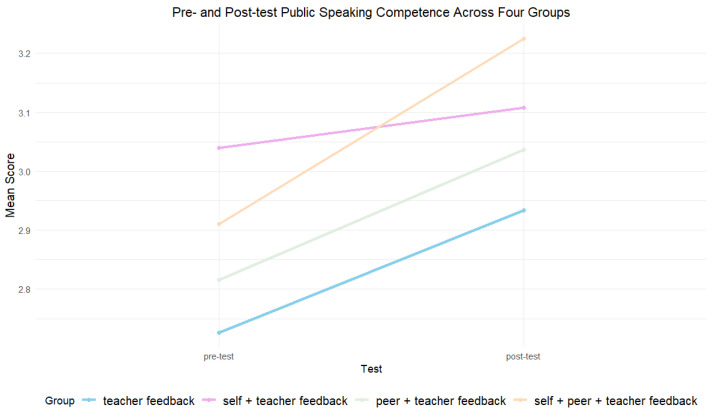
Pre- and post-test public speaking competence across four groups.

The paired sample *t*-test showed that there was significant improvement from pre-test for Group 1 (*t*(24) = 3.888, *p* = 0.001, Cohen’s *d* = 0.778), Group 3 (*t*(24) = 5.078, *p* < 0.001, Cohen’s *d* = 1.02), and Group 4 (*t*(23) = 5.497, *p* < 0.001, Cohen’s *d* = 1.12). However, the improvement for Group 2 was not significant (*t*(22) = 1.357, *p* = 0.188, Cohen’s *d* = 0.277) (see Figure 7). A significant difference in the students’ gain scores across groups was identified (*F*(3, 94) = 3.889, *p* = 0.011, partial η^2^ = 0.110), indicating that the feedback source explained 11% of the variance. The post hoc analysis revealed that Group 4 made significantly more improvements compared to Group 2 (*p* = 0.006).

### 3.5. Correlation between Students’ Feedback Engagement and Public Speaking Performance

The Pearson correlation revealed a weak correlation between the gain of students’ public speaking competence and the gain of their behavioral engagement (*r* = 0.210, *p* = 0.038) as well as with the gain of their total engagement with feedback (*r* = 0.233, *p* = 0.021). However, no correlation was observed between students’ public speaking competence and their cognitive engagement (*r* = 0.189, *p* = 0.063) or emotional engagement (*r* = 0.148, *p* = 0.146). Additionally, a moderate correlation was identified between the three types of engagement (see Table 5).

## 4. Discussion

This study aimed to investigate how the three feedback sources impact learners’ engagement with feedback and their public speaking competence using validated PSFES responses and public speaking ratings. For RQ1, regarding the impact of the three feedback sources on student engagement, Group 3 (peer and teacher feedback) was significantly more engaged than Group 2 (self- and teacher feedback) cognitively, behaviorally, and emotionally, as well as being more engaged than Group 4 (all feedback sources) emotionally. Group 3 made significant improvement in behavioral, emotional, and total engagement, with the increase in cognitive engagement approaching significance. In this study, peer feedback was preceded by a group discussion and then further elaborated upon by the teacher. Concurring with Banister [5], group endorsement in discussions enhances students’ confidence, and group-based peer feedback helps alleviate concerns of power dynamics that may arise in one-on-one peer feedback scenarios; thus it tends to be more emotionally engaging. During the group discussion and the following verbal peer feedback process, students would express uncertainties and different opinions, often seeking the teacher’s decisive input to clarify points and resolve disputes. Then, feedback from teachers, characterized as authoritative, credible, and motivating [3,10], confirmed students’ comments and addressed the disputes among students. This sufficient exchange of ideas allowed students to engage in deeper cognitive processes, paying attention to and understanding the points brought up by different parties, thus becoming better aware of the gaps between their current behaviors and desired performance. This cognitive awareness then translated into tangible actions, such as taking notes, making revisions, and practicing speech delivery.

For the comparison between Group 3 and Group 1, the combination of peer and teacher feedback did not prove to be more cognitively engaging than teacher feedback alone. Both groups showed significant improvements in behavioral engagement. This underscores the crucial role of teachers, who typically provide more in-depth and systematic feedback, as highlighted in van Ginkel et al. [21], in guiding and motivating students to act upon it. However, peer and teacher feedback combined seems to be more emotionally engaging as it is perceived to be more useful and anticipated by students compared to teacher feedback alone. Teacher feedback, when combined with peer feedback, can reduce the power dynamics and negative emotional responses commonly associated with teacher feedback [48]. Additionally, this integration resulted in students’ better awareness of their strengths and weaknesses, leading to more academic endeavors such as note-taking, revision, structure organization, evidence searching, and delivery practice. Peer feedback, delivered in more relatable language [12], encourages more revisions and fosters students’ sense of ownership of their work [48].

Another notable pattern in our findings suggests that the self-feedback, when conducted in a plenary class, can have negative effects on student engagement. Group 2 experience significant declines in cognitive, emotional, and total engagement, and Group 4 showed significant drop in their emotional engagement. In our study, self-feedback was succeeded by teacher feedback in a plenary class following the practice of an award-winner in a case study by Carless [6] in the hope of establishing the dialog between the teacher and the speakers. Several reasons may explain for this result. Cognitively, as self-feedback may not be as constructive or actionable as feedback from peers or teachers. Students might not know how to translate their self-observations into specific strategies for improvement [17,49], leading to stagnation or decline in engagement. Regarding the emotional engagement, first, the negative emotional impact of self-feedback in this study may be attributed to the repetitive nature of self-critique in class followed by the subsequent written self-critique after class, which can cause students to be overly critical of themselves [17]. Second, in a context of Confucian heritage where face-saving is a salient feature [50], explicit self-critique in plenary class can be uninviting, particularly when the students are novice speakers. Third, we prioritized self-feedback at the beginning of the process, aiming for students to independently identify strengths and weaknesses, which was intended to increase the relevance and impact of subsequent peer and teacher feedback [49]. However, this approach may have inadvertently heightened students’ anxiety, leading to a suppression of their emotional engagement. Starting with peer feedback might help ease students into the feedback process, making them more open to subsequent teacher feedback. The lack of specific actionable strategies, coupled with emotional discomfort may have caused the deterioration and reduction in behavioral engagement.

It should be noted that the present study did not consider the order effect of feedback sources. We designed teacher feedback to be followed by self- or peer feedback based on the practice of previous studies [4,5,6], aiming to motivate students to engage in the feedback dialog. However, presenting teacher feedback before student feedback might serve as a feedback model for students to emulate, stimulating their reflective processes and setting the stage for their own feedback, thus are more cognitively engaging. Additionally, as the teacher feedback may allow students more time to organize their thoughts, reducing anxiety and enhancing emotional engagement.

Regarding RQ2 about the effect of different feedback sources on students’ public speaking performance, significant improvements were observed in all groups except Group 2. Group 3 and 4 showed comparable improvement; however, the slight advantage of Group 4 made it significantly more effective compared to the limited improvement in Group 2. The improvement in public speaking competence observed in Group 4 could be attributed to the benefit they derived from the additional peer feedback process, as discussed earlier. Furthermore, Nicol et al. [51] posited that peer feedback could have triggered a reflective process wherein students utilize feedback provided to their peers to improve their own work, even without being asked to. Given the possible self-initiated evaluation during peer feedback and the post-class self-feedback required of Group 4, overt in-class self-critique does not appear to be necessary.

Regarding RQ3, this study found that students’ public speaking competence was positively correlated with their total engagement and behavioral engagement, while showing no significant correlation with their emotional or cognitive engagement, which was different from Zheng and Yu [31]. Group 4 demonstrated the greatest improvement in public speaking competence, showing enhanced behavioral engagement despite decreases in emotional and cognitive engagement. Benefiting from additional peer feedback, Group 4 likely offset any emotional discomfort by increasing their participation and involvement in the task, as this group made the most revisions among the four groups. This study suggests that learners’ cognitive and emotional states tend to stabilize over time, and effective teaching and learning are ultimately demonstrated through tangible behaviors.

## 5. Conclusions

It should be acknowledged that the sample size for PSEFS validation was relatively small, although Rasch analysis is robust with small sample sizes [37]. Future research can validate the PSEFS using larger sample sizes. Additionally, throughout the intervention, each student received just one individualized feedback due to practical challenges with the class’s time constraints, although all students received feedback on speeches from other students on four different topics. Further research may consider extending the duration of the intervention and increasing the frequency of feedback loops to examine the replicability of the observed effects. Furthermore, this study did not account for the order effect of the feedback sources, an important variable that can be systematically manipulated in future research.

This study found that peer and teacher feedback combined tend to engage students cognitively, behaviorally, and emotionally. However, the introduction of self-feedback in plenary classes may not have the desired effects due to emotional discomfort. second, the incorporation of self-, peer, and teacher feedback resulted in the best public speaking performance. Third, students’ public speaking performance is correlated with their behavioral and overall engagement. For practitioners, it is suggested that peer feedback as a group should be preceded by group discussion to enhance pooled credibility and be followed by teacher feedback to validate and complement peer feedback, whereas self-feedback be conducted after class at students’ comfort to enhance students’ engagement and public speaking performance.

## Figures and Tables

**Table 1:** Rasch–Andrich RSM of Public Speaking Feedback Engagement Scale (PSFES).

Test	Subscale	1st Contrast	Person Separation	Person Reliability	Item Separation	Item Reliability	Item Infit MnSq	Item Outfit MnSq
Pre	Cognitive	1.80	1.57	0.71	1.53	0.70	0.80–1.20	0.80–1.15
	Behavioral	1.48	1.35	0.65	5.79	0.97	0.70–1.23	0.69–1.09
	Emotional	1.79	1.28	0.62	3.41	0.92	0.74–1.48	0.67–1.56
Post	Cognitive	1.77	1.67	0.74	2.51	0.86	0.78–1.24	0.82–1.18
	Behavioral	1.58	1.51	0.70	5.68	0.97	0.81–1.27	0.82–1.29
	Emotional	1.64	1.15	0.57	2.65	0.88	0.95–1.05	0.90–1.07

MnSq = mean square.

**Table 2:** Many-facet Rasch measurement of the ratings.

Test	Observed Average	Fair Average	Measure (*logits*)	Model SE	Infit MnSq	Outfit MnSq
Pre	2.03–3.66	2.02–3.67	−2.92–1.69	0.18–.21	0.57–1.47	0.58–1.45
Post	1.71–4.05	1.72–4.05	−4.32–3.30	0.18–.22	0.54–1.47	0.54–1.45

**Table 3:** Descriptive statistics of gain scores of engagement for four groups.

	Sample Size	Cognitive Engagement		Behavioral Engagement		Emotional Engagement		Total Engagement	
	N	M	SD	M	SD	M	SD	M	SD
Group 1	25	1.16	3.94	1.56	3.51	0.40	1.78	3.12	7.74
Group 2	24	−2.29	2.16	−0.46	2.64	−1.00	1.02	−3.75	3.77
Group 3	25	1.04	2.57	2.60	3.40	1.04	2.28	4.68	6.33
Group 4	24	−1.17	3.58	0.71	3.01	−0.96	1.99	−1.42	6.50

M = mean; N = number; SD = standard deviation.

**Table 4:** Paired sample *t*-test for engagement of four groups.

Group	M	SD	*t*-Value	*df*	*p*-Value	Cohen’s *d*
*Cognitive engagement*						
Group 1	1.16	3.94	1.471	24	0.154	0.294
Group 2	−2.29	2.16	−5.206	23	<0.001 ***	−1.063
Group 3	1.04	2.57	2.021	24	0.055	0.404
Group 4	−1.17	3.58	−1.595	23	0.124	−0.326
*Behavioral engagement*						
Group 1	1.56	3.51	2.22	24	0.036 *	0.444
Group 2	−0.46	2.64	−0.851	23	0.403	−0.174
Group 3	2.60	3.40	3.82	24	0.001 **	0.764
Group 4	0.71	3.01	1.151	23	0.261	0.235
*Emotional engagement*						
Group 1	0.40	1.78	1.124	24	0.272	0.225
Group 2	−1.00	1.02	−4.796	23	<0.001 ***	−0.979
Group 3	1.04	2.28	2.279	24	0.032 *	0.456
Group 4	−0.96	1.99	−2.361	23	0.027 *	−0.482
*Total engagement*						
Group 1	3.12	7.74	2.015	24	0.055	0.403
Group 2	−3.75	3.77	−4.876	23	<0.001 ***	−0.995
Group 3	4.68	6.33	3.697	24	0.001 **	0.739
Group 4	−1.42	6.50	−1.068	23	0.297	−0.218

* *p* < 0.05, ** *p* < 0.01, *** *p* < 0.001; M = mean difference (post-test minus pre-test); SD = standard deviation.

**Table 5:** Pearson correlation between engagement with feedback and public speaking competence.

	PSC	TE	CE	BE	EE
PSC	-				
TE	0.233 *	-			
CE	0.189	0.835 ***	-		
BE	0.210 *	0.816 ***	0.445 ***	-	
EE	0.148	0.734 ***	0.485 ***	0.452 ***	-

* *p* < 0.05, *** *p* < 0.001; BE = behavioral engagement; CE = cognitive engagement; EE = emotional engagement; PSC = public speaking competence; TE = total engagement.

## Data Availability

The data presented in this study are available on reasonable request from the corresponding author due to ethical reasons.

## Appendix A

### Appendix A.1. Public Speaking Feedback Engagement Scale (PSFES)

**Public Speaking Feedback Engagement Scale (PSFES)**	
Directions: Please indicate in the following scale to what extent you agree with the following statements (1 = strongly disagree; 2 = disagree; 3 = not sure/neutral; 4 = agree; 5 = strongly agree) and provide your complementary comment. *Cognitive engagement*	
1. I pay attention to the feedback.	(1) (2) (3) (4) (5)
2. I understand the feedback.	(1) (2) (3) (4) (5)
3. I reflect on and evaluate the feedback.	(1) (2) (3) (4) (5)
4. I realize my strengths and weaknesses through the feedback.	(1) (2) (3) (4) (5)
5. I strategize to improve my speech based on the feedback.	(1) (2) (3) (4) (5)
6. I monitor myself against the feedback when I prepare the next speech.	(1) (2) (3) (4) (5)
*Behavioral engagement*	
7. I take careful notes of the feedback.	(1) (2) (3) (4) (5)
8. I discuss the feedback with my teacher and classmates after class.	(1) (2) (3) (4) (5)
9. I make revisions based on the feedback.	(1) (2) (3) (4) (5)
10. I mark the structure of my next speech based on the feedback.	(1) (2) (3) (4) (5)
11. I search relevant sources for evidence to support my point.	(1) (2) (3) (4) (5)
12. I practice delivering my next speech based on the feedback.	(1) (2) (3) (4) (5)
*Emotional engagement*	
13. I enjoy receiving/providing feedback.	(1) (2) (3) (4) (5)
14. I find the feedback helpful and valuable.	(1) (2) (3) (4) (5)
15. I look forward to the feedback on my next speech.	(1) (2) (3) (4) (5)
*Other comments*	

### Appendix A.2. Feedback Sheet

**Feedback Sheet**
Name: ____________ Evaluator ____________________ Please give your comment on the speaker’s performance based on the following guidelines.
*The Speech Opening*
1. Did the speech open with a story, a joke, a startling statistic, a controversial statement, a quotation?
2. Was the speech’s thesis statement or intent clearly presented?
3. Was there a clear transition from introduction to body?
*The Speech Body*
4. Were the main points clear?
5. Were examples or statistics provided to support the arguments?
6. Was the speech organized logically? Was it easy to follow?
7. Did the speaker transit smoothly from one point to another?
*The Speech Conclusion*
8. Was the thesis stressed or main points reviewed in the conclusion?
9. Was the conclusion memorable?
10. If appropriate, was there a call-to-action?
*Delivery Skills*
11. Was the speaker loud enough to be heard clearly?
12. Were loud and soft variations used appropriately?
13. Were the slow and fast pace variations used appropriately?
14. Were pauses used to aid understandability, heighten excitement, or provide drama?
15. Did the speaker articulate clearly?
16. Did the speaker’s postures display confidence and poise?
17. Was eye contact effective in connecting the speaker to the audience?
*Global Comment*
18. Were you convinced?

### Appendix A.3. Fit Statistics of PSFES Items

**Subscale**		**Pre-Test**		**Post-Test**	
	**Item**	**Infit MnSq**	**Outfit MnSq**	**Infit MnSq**	**Outfit MnSq**
Cognitive	1	0.85	0.89	1.12	1.07
	2	1.1	0.98	0.78	0.82
	3	0.8	0.8	0.85	0.88
	4	1.2	1.15	1.24	1.18
	5	0.96	0.87	0.94	0.92
	6	1.03	0.95	1.09	1.1
Behavioral	7	0.7	0.69	0.86	0.87
	8	1.09	1.18	1.27	1.29
	9	0.96	0.94	0.96	0.97
	10	1.16	1.11	0.9	0.88
	11	1.23	1.09	1.16	1.12
	12	0.98	0.98	0.81	0.82
Emotional	13	0.73	0.78	1.05	1.07
	14	0.74	0.67	0.98	0.9
	15	1.48	1.56	0.95	0.9

### Appendix A.4. Rating Scale Model’s Category Structure for PSEFS

**Test**	**Category** **Level**	**Observed** **Average**	**Expected** **Average**	**Infit** **MnSq**	**Outfit** **MnSq**	**Andrich** **Threshold**	**Category** **Measure**
Pre	Cognitive						
	2	−0.97	−0.87	0.92	0.79	NONE	−2.71
	3	−0.21	−0.17	0.95	0.88	−1.02	−1.57
	4	0.96	0.93	1	1.04	1.64	0.67
	5	2.53	2.58	1.1	0.99	2.66	3.77
	Behavioral						
	1	−0.62	−0.82	1.13	1.02	NONE	−4.39
	2	−0.19	−0.18	1	0.97	−3.27	−1.57
	3	0.51	0.53	0.85	0.74	−0.54	0.15
	4	1.36	1.36	1.13	1.19	0.17	1.63
	5	2.44	2.42	0.89	0.97	2.91	4.04
	Emotional						
	2	−3.05	−2.89	0.81	0.64	NONE	−4.52
	3	−0.9	−0.94	1.03	1.15	−3.31	−2.46
	4	2.17	2.18	0.93	0.96	−1.62	1.66
	5	5.45	5.44	0.99	1	4.92	6.02
Post	Cognitive						
	2	−0.71	−0.82	1.04	1	NONE	−4.22
	3	0.14	0.06	1.09	1.13	−3.07	−1.67
	4	1.47	1.54	0.99	0.95	−0.26	1.55
	5	3.5	3.42	0.91	0.91	3.34	4.46
	Behavioral						
	1	−2.06	−1.2	0.6	0.74	NONE	−5.63
	2	−0.4	−0.25	0.89	0.9	−4.51	−2.78
	3	1.06	0.98	1.03	1.03	−1.01	0.08
	4	2.42	2.43	1.01	0.98	1.17	2.78
	5	4.07	4.09	1.05	1.03	4.35	5.48
	Emotional						
	2	−1.7	−2.12	1.18	1.04	NONE	−4.56
	3	−0.35	−0.36	1.06	1.02	−3.41	−2.08
	4	2.07	2.12	1	1.03	−0.75	1.71
	5	4.31	4.24	0.91	0.85	4.16	5.26

### Appendix A.5. Fit Statistics of the Ratings for the Pre- and Post-Tests

**Student No.**	**Pre-Test**		**Post-Test**	
	**Infit MnSq**	**Outfit MnSq**	**Infit MnSq**	**Outfit MnSq**
1	1.01	1.01	1.14	1.14
2	0.91	0.91	0.87	0.85
3	1.3	1.3	0.62	0.61
4	0.97	0.97	0.85	0.85
5	1.03	1.03	1.17	1.16
6	1.07	1.08	1.31	1.31
7	1.03	1.04	1.04	1.04
8	0.79	0.79	0.93	0.93
9	0.91	0.91	1.43	1.42
10	0.83	0.83	0.92	0.92
11	0.95	0.95	1.09	1.1
12	0.9	0.91	0.75	0.76
13	1.36	1.35	1.25	1.24
14	1.28	1.26	1.11	1.11
15	1.13	1.13	1.39	1.37
16	0.97	0.96	0.71	0.7
17	1.2	1.2	1.28	1.26
18	1.06	1.06	1.17	1.16
19	1.42	1.41	0.86	0.86
20	1.22	1.22	0.93	0.93
21	1.47	1.45	1.12	1.11
22	1.18	1.18	0.76	0.75
23	0.97	0.97	0.75	0.75
24	0.96	0.96	1.1	1.08
25	1.26	1.27	1.19	1.19
26	1.19	1.19	1.43	1.43
27	0.74	0.74	1.3	1.3
28	1.09	1.09	1.23	1.23
29	1.06	1.06	0.97	0.98
30	1.01	1.01	1.18	1.17
31	1.1	1.1	1.18	1.17
32	0.93	0.93	0.89	0.89
33	1.17	1.18	0.74	0.72
34	0.88	0.88	0.93	0.93
35	0.67	0.67	1.19	1.17
36	1.2	1.19	0.92	0.92
37	1.1	1.1	1.04	1.04
38	0.98	0.99	0.78	0.78
39	1.19	1.19	0.9	0.9
40	1.31	1.32	1.33	1.32
41	0.79	0.79	0.88	0.86
42	0.7	0.71	0.83	0.83
43	1.24	1.23	1.44	1.43
44	0.88	0.88	0.81	0.8
45	0.91	0.91	0.79	0.77
46	1.12	1.12	1.18	1.16
47	1.03	1.03	1.12	1.13
48	0.9	0.9	0.73	0.74
49	1.01	1.01	1.47	1.45
50	0.87	0.87	0.95	0.94
51	0.82	0.82	1.05	1.04
52	1.2	1.2	0.71	0.7
53	1.17	1.17	1.22	1.21
54	1.13	1.13	0.93	0.91
55	0.74	0.74	0.54	0.54
56	0.96	0.95	0.98	0.98
57	0.99	0.99	0.57	0.56
58	0.98	0.98	0.79	0.79
59	0.86	0.86	0.83	0.83
60	1.07	1.06	0.7	0.69
61	0.75	0.74	1.13	1.14
62	0.91	0.92	1.33	1.33
63	0.63	0.63	0.96	0.96
64	0.73	0.73	1.12	1.11
65	1.12	1.12	0.56	0.56
66	1	1	1.07	1.07
67	1.07	1.07	0.87	0.87
68	1	0.99	0.71	0.71
69	0.84	0.84	0.62	0.63
70	0.65	0.66	1.03	1.02
71	0.83	0.84	0.77	0.76
72	0.73	0.74	0.77	0.76
73	1.09	1.08	0.98	0.97
74	1.13	1.12	0.9	0.9
75	0.89	0.89	0.94	0.94
76	1.08	1.08	1.37	1.37
77	1.02	1.02	1.13	1.13
78	0.91	0.91	0.97	0.97
79	0.95	0.95	1.29	1.29
80	0.83	0.83	1.36	1.37
81	1.05	1.05	0.63	0.61
82	1.24	1.24	0.8	0.8
83	1.12	1.13	1.28	1.27
84	1.02	1.02	0.77	0.78
85	0.92	0.93	1.1	1.11
86	1.05	1.05	1.09	1.09
87	0.57	0.58	0.96	0.96
88	0.87	0.87	0.72	0.73
89	0.82	0.82	0.95	0.95
90	1.16	1.16	1.05	1.05
91	0.95	0.95	0.96	0.97
92	0.85	0.85	1.43	1.42
93	1.17	1.16	0.66	0.66
94	1.12	1.13	1.1	1.13
95	0.78	0.78	1.04	1.04
96	0.97	0.97	1.35	1.35
97	1.02	1.02	1.21	1.21
98	0.97	0.97	0.67	0.67

### Appendix A.6. Descriptive Statistics of Engagement and Public Speaking Competence for Four Groups in Pre-Test and Post-Test

		**Cognitive** **Engagement**		**Behavioral** **Engagement**		**Emotional** **Engagement**		**Total** **Engagement**		**Public Speaking** **Competence**	
	**Group**	**Pre**	**Post**	**Pre**	**Post**	**Pre**	**Post**	**Pre**	**Post**	**Pre**	**Post**
N	Group 1	25	25	25	25	25	25	25	25	25	25
	Group 2	24	24	24	24	24	24	24	24	24	24
	Group 3	25	25	25	25	25	25	25	25	25	25
	Group 4	24	24	24	24	24	24	24	24	24	24
Mean	Group 1	24	26	21.8	23.4	13.6	14	60.2	63.4	2.73	2.93
	Group 2	25.50	23.3	22.7	22.2	13.8	12.8	62	58.2	3.04	3.11
	Group 3	23.30	24.4	20	22.6	12.4	13.4	55.6	60.3	2.82	3.04
	Group 4	24.40	23.2	20.8	21.5	13.5	12.5	58.6	57.2	2.91	3.22
SD	Group 1	3.22	2.66	3.16	2.94	1.22	1.38	6.19	5.38	0.327	0.42
	Group 2	2.65	2.49	3.34	2.99	1.26	1.26	6.24	5.96	0.243	0.289
	Group 3	2.63	2.43	2.86	2.43	1.8	1.55	6.12	4.31	0.345	0.366
	Group 4	2.53	2.96	3.25	2.83	1.74	2	5.83	6.83	0.281	0.318
Skewness	Group 1	−0.54	−0.677	−0.05	−0.374	−0.473	−1.13	−0.257	−0.452	−0.203	−0.844
	Group 2	0.09	1.18	0.492	0.917	−0.338	0.801	0.477	1.17	−0.81	−1.11
	Group 3	−1.60	−0.174	−0.118	−0.305	0.108	−0.882	−0.327	0.642	0.306	0.676
	Group 4	−0.06	0.293	0.003	0.293	−0.621	−0.267	−0.008	0.237	−0.405	0.325
Kurtosis	Group 1	−0.30	0.799	−0.602	−0.961	−0.892	−0.122	−0.898	−0.016	−0.578	1.48
	Group 2	−0.35	1.23	−0.428	1.24	−1.6	−0.521	−0.656	1.28	0.593	2.36
	Group 3	3.40	−0.644	−0.502	−0.154	−0.416	1.01	−0.367	−0.129	1.53	0.54
	Group 4	−1.27	−0.795	−1.34	0.156	−1.21	−1.15	−1.16	−0.192	−0.63	1.19
N = number; Post = post-test; Pre = pre-test; SD = standard deviation.											
